# Combined Brilliant and Trypan Blue As Vital Dyes for Chromovitrectomy

**DOI:** 10.18502/jovr.v19i2.7652

**Published:** 2024-06-21

**Authors:** Bruno Fortaleza de A. Ferreira, Gustavo Sakuno, Juliana Mika Kato, Rodolfo Bonatti, Álvaro Fernandes Ferreira, Aloisio Fumio Nakashima, Yoshitaka Nakashima

**Affiliations:** ^1^Department of Ophthalmology, University of Sao Paulo, Sao Paulo, Brazil; ^2^Department of Ophthalmology, Hospital Geral de Fortaleza, Ceará, Brazil; ^4^Bruno Fortaleza de A. Ferreira: https://orcid.org/0000-0003-3555-9122

**Keywords:** Brilliant Blue, Trypan Blue, Vitrectomy

## Abstract

Brilliant blue 0.05% and trypan blue 0.1% were mixed in a proportion of 1:1 in a 1-mL syringe. This combination produced a waterfall effect with the fast sinking of the dye to the posterior pole and little diffusion through the vitreous cavity. Therefore, it can effectively stain the internal limiting membrane and the epiretinal membrane with a good contrast during surgeries for a macular hole, myopic foveoschisis, and macular pucker.

##  INTRODUCTION

Vital dyes must balance staining strength, toxicity, and cost.^[1]^ Brilliant Blue (BB) has a good affinity for the internal limiting membrane (ILM), but it often diffuses into the vitreous cavity instead of depositing onto the retinal surface.^[2,3]^ Polyethylene glycol (PEG) and 50% glucose are known densifying vehicles for BB; however, PEG is not always available in most surgical centers, and both can induce hyperosmolar stress in retinal pigmented epithelial cells.^[3,4,5]^ We describe a feasible way to improve chromovitrectomy using a combined solution of BB and trypan blue (TB) for staining and removal of the macular membrane (ERM) during pars plana vitrectomy.

**Table 1:** Comparison of osmolarity and pH between balanced salt solution and vital dyes


**Solution**	**Osmolarity (mOsm)**	**pH**
Balanced salt solution	300	7.5
Trypan blue (TB) 0.1%	319	7.45
Brilliant blue (BB) 0.05%	290	7.4
1:1 BB 0.05% + TB 0.1%	304.1	7.09
	
	

**Figure 1:**
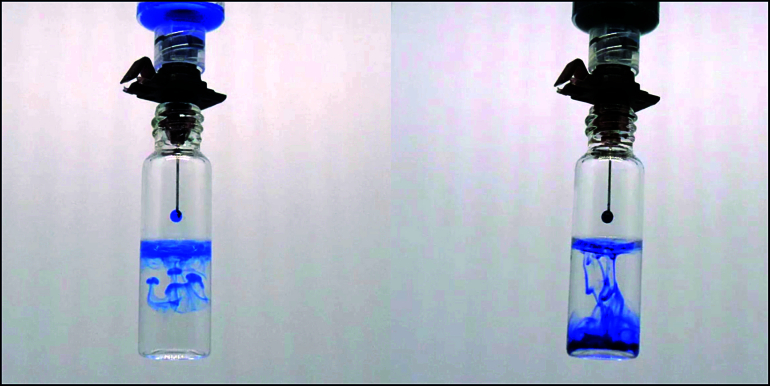
Brilliant blue (on the left) and brilliant trypan (on the right) drops behavior in balanced salt solution.

**Figure 2:**
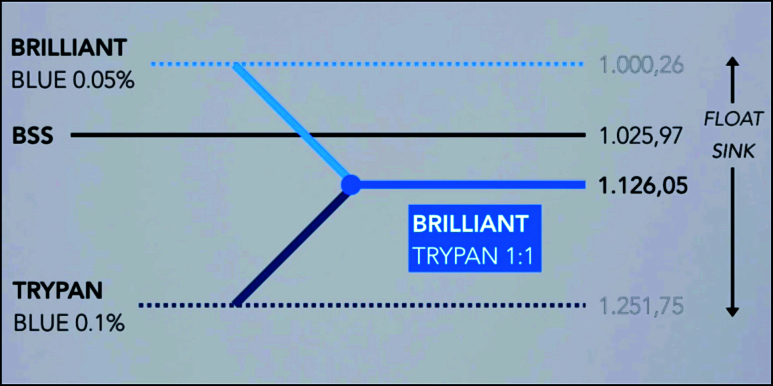
Comparison of brilliant blue 0.05%, balanced salt solution (BSS), trypan blue 0.1%, and brilliant trypan densities.

**Figure 3:**
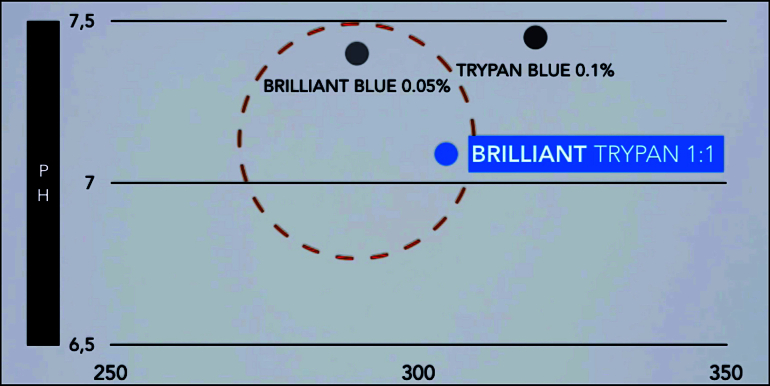
Comparison of pH and osmolarity between brilliant trypan and other vital dyes, showing values within the safety range (about 7 and 290, respectively).

**Figure 4:**
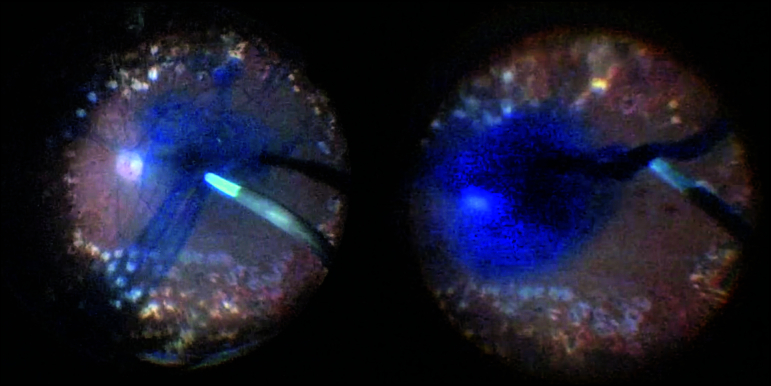
Diffusion of brilliant blue through balanced salt solution (on the left) and “waterfall effect” produced by combination of brilliant and trypan blue (on the right) during vitrectomy.

##  SURGICAL METHOD 

BB 0.05% (Ophthalmos, Sao Paulo, Brazil) and TB 0.1% (Ophthalmos, Sao Paulo, Brazil) were mixed in a proportion of 1:1 in a 1-mL syringe. Previous laboratory tests revealed that this solution instantly sank into a balanced salt solution [Figure 1]. This mixed solution has a density of 1126.05 mg/ml [Figure 2], an osmolarity of 304.1 mOsm, and a pH value of 7.09 [Figure 3]. After core vitrectomy and inducing posterior vitreous detachment, 0.2 mL of the combined solution was slowly injected into the vitreous cavity through a dual-bore cannula. At this point, if non-valved trocars were used, BSS infusion must be temporarily turned off to avoid whirlwinds (this is not necessary with valved trocars). Like PEG solutions, the combination of BB and TB produced a waterfall effect with the fast sinking of the dye to the posterior pole without diffusion through the vitreous cavity [Figure 4]. Within five sec, we aspirated the dye out of the cavity and observed effective ILM and ERM staining. This step was followed by successful ERM and ILM removal.

##  RESULTS

In three cases with different pathologies (macular hole, myopic foveoschisis, and macular pucker), the ERM and the ILM were successfully identified by a bluish stain and completely removed. In addition, the combined BB and TB solution was used to stain the anterior capsule when phacoemulsification was performed without intraoperative complications. All patients presented good anatomical response during a month of follow-up, with no signs of toxicity related to the dye, such as retinal edema, pigmentary changes, or atrophy on OCT.

##  DISCUSSION

The combination of BB and TB in a 1:1 proportion has a higher density than BB or TB alone, reaching the posterior pole in a shorter time. Additionally, it presents pH and osmolarity within the safety range [Table 1]. Previous histopathological toxicity studies also revealed no significant morphological changes in the retina.^[6]^ In this series of three cases, the solution improved efficiency in ERM and ILM identification and removal, acting as a high-density dye with good staining strength. Compared to commercially dual-combination solutions, which may not be available, this combined solution of BB and TB is an alternative with similar laboratory properties. It also allows using a single dye syringe during phacovitrectomy surgery, as it stains the anterior lens capsule with no additional toxicity.

##  Financial Support and Sponsorship

None.

##  Conflicts of Interest

None.
